# Experimental tests of habitat preferences in the Comal Springs dryopid beetle, *Stygoparnus comalensis*

**DOI:** 10.7717/peerj.20507

**Published:** 2025-12-16

**Authors:** Matthew R. Pintar

**Affiliations:** BIO-WEST, San Marcos, TX, United States of America

**Keywords:** Aquatic beetle, Dryopoidea, Habitat selection, Phototaxis, Spring ecosystems, Stygobitic beetle

## Abstract

The Comal Springs dryopid beetle, *Stygoparnus comalensis* Barr and Spangler (Coleoptera: Dryopidae), is a federally endangered species endemic to three spring systems in central Texas, United States. Improving our understanding of the biology of this species is necessary for making progress towards the goals of its protection: establishing a monitoring program and maintaining captive reproducing populations. A series of experiments were conducted in captivity to empirically examine habitat preferences of *S*. *comalensis* while using a similar co-occurring species, *Stenelmis sexlineata* Sanderson, for comparison. *Stygoparnus comalensis* had a strong affinity for wood over leaves and rocks and had fuller guts when offered wild-conditioned wood compared with captive-conditioned wood. Adults and larvae of *S*.* sexlineata* were also attracted to wood when offered rocks, but did not prefer wood when also offered leaves. Both species responded to light, but responses were light- and species-specific, with *S. comalensis* avoiding shorter wavelength light and being attracted to longer wavelength light whereas *S*. *sexlineata* seemed to hide from all light types. Both species were attracted to conspecifics but not heterospecifics. *Stygoparnus comalensis* did not exhibit any clear response to flowing water (*S*. *sexlineata* was not examined). These results provide insight into the potential adaptations of a spring-endemic beetle species and environmental relationships that can potentially be used for improving monitoring and conservation of wild populations.

## Introduction

The Comal Springs dryopid beetle, *Stygoparnus comalensis* Barr and Spangler (Coleoptera: Dryopidae) is a beetle that is known to primarily occur in the Comal Springs system, Comal County, Texas, USA; it has also been found in two springs in neighboring Hays County, Texas (Gibson, Harden & Fries, 2008). Comal Springs has historically been the largest spring complex in Texas, consisting of numerous small spring openings emanating from the limestone along the fault at the base of the Balcones Escarpment (Crowe & Sharp Jr., 1997; Brune, 2002). Water temperature (23.4 °C) and other physiochemical parameters of the water coming from the aquifer are stable year-round (Guyton, 1979), although springflow may cease at higher elevation spring openings when aquifer levels drop (BIO-WEST, 2024). *Stygoparnus comalensis*, along with several other species endemic to the ecosystem, were listed as endangered by the US Fish and Wildlife Service in 1997 (United States Fish and Wildlife Service, 1997). The primary threat to these species is considered to be declining springflows due to overextraction of water from the aquifer and drought, though other potential risks such as groundwater contamination are recognized (Hutchins, 2018; United States Fish and Wildlife Service, 2024). The Edwards Aquifer Habitat Conservation Plan (EAHCP, 2012) has the goals of protection, monitoring, and establishment of self-propagating populations of listed species, but due to the rarity of *S*. *comalensis* (Gibson, Harden & Fries, 2008; Huston et al., 2015), its efforts have lagged behind other species.

Much of what is known regarding *S*. *comalensis* is a result of general work in the Comal Springs system and work conducted with the co-occurring endangered spring-endemic species *Heterelmis comalensis* Bosse, Tuff, and Brown (Coleoptera: Elmidae). Studies of the distribution of both species have found *S*. *comalensis* at much lower rates than *H*. *comalensis* (Gibson, Harden & Fries, 2008), and during biomonitoring efforts, *H*. *comalensis* occurs at a rate >300 times higher than *S*. *comalensis* (BIO-WEST, 2024). Techniques used for captive propagation of *H*. *comalensis* (United States Fish and Wildlife Service, 2022) have been used as a basis for work on *S*. *comalensis*, and studies of the biology of *H*. *comalensis* often include *S*. *comalensis* since they can be collected together (Huston et al., 2015; Lucas et al., 2016; Kosnicki, 2019; Nair, Diaz & Nowlin, 2021).

Only a few aspects of the biology and ecology of *S*. *comalensis* have been assessed. Within the Comal Springs system, *S*. *comalensis* have most commonly been found in a locality that is surrounded by sycamore trees (*Platanus* spp.)—an area that has also maintained springflow through recent low-flow periods and has a mix of substrate sizes ranging from silt to cobble, but not dominated (at the surface) by bedrock (BIO-WEST, 2025, unpublished data). While observations indicate the potential occurrence of *S*. *comalensis* near sycamore roots (BIO-WEST, 2022), this and other environmental relationships have not been quantified. Adult *S*. *comalensis* have been collected primarily from near-surface habitats, although their vestigial eyes and undeveloped wings have led to the suggestion that they are a subsurface species (Barr & Spangler, 1992; Gibson, Harden & Fries, 2008; Shepard, 2019). When held in captivity, wild-caught adults have survived for as long as 21 months (Barr & Spangler, 1992), and limited captive breeding efforts have indicated they have long larval stages that take over one year to reach the adult stage (BIO-WEST, 2022). Studies in captivity of the lengths of the three immature life stages (egg, larva, pupa) have also shown low rates of egg laying by females, low rates of egg hatching, and even lower rates of pupation (BIO-WEST, 2022).

More information is needed regarding the habitat preferences, environmental associations, and biology of *S*. *comalensis* to make progress towards the goals of the Edwards Aquifer Habitat Conservation Plan (EAHCP). Given the challenges associated with the wide range of environmental conditions and the limited ability to detect and collect *S*. *comalensis* in the wild, experiments in controlled conditions can be used to target responses of *S*. *comalensis* to specific environmental characteristics. Because of the vestigial eyes on *S*. *comalensis* and its endemicity to spring habitats, a similarly sized, co-occurring, more geographically widespread, and relatively closely related species, *Stenelmis sexlineata* (Elmidae), was included in experiments to contextualize the responses of *S*. *comalensis*. A series of laboratory microcosm experiments were conducted with these beetles in captivity to determine their responses to physical habitat structure, light exposure, water flow, presence of other beetles, and variation in food quality. Due to limitations on numbers of beetles available for study, these experiments aimed to obtain a general understanding of the responses by *S*. *comalensis* to these factors rather than assess any given factor in greater detail. These experiments were conducted with the aim of gaining a better understanding the basic biology of *S*. *comalensis* across a range of topics that could be used to inform further efforts to protect it.

## Materials & Methods

### General experimental design

In most of the following experiments, adult *S*. *comalensis* were tested alongside adults and larvae of *Stenelmis sexlineata* Sanderson (Coleoptera: Elmidae), a non-threatened beetle that co-occurs as both adults and larvae with *S*. *comalensis*. Larvae of *S*. *comalensis* were not included as none were found during the study period. The inclusion of *S*. *sexlineata* primarily served to provide a contrast to the habitat preferences of *S*. *comalensis* as it is the most similar species morphologically in Comal Springs to *S*. *comalensis*. While both species should not be expected to have the same habitat preferences, the comparison is pertinent to contextualize the responses of *S*. *comalensis*. Adults of both species are relatively similar in body size (approximately three mm body length), though *S*. *sexlineata* has developed eyes, some populations are initially capable of flight upon pupation (White, 1978; Zera & Denno, 1997; Shepard, 2019), and is widespread across the central United States (Schmude, 1992). They are both relatively closely related taxonomically being in the superfamily Dryopoidea (Hayashi et al., 2024); no other species of Dryopidae occur in the system except as vagrants. Although competitive interactions among the species during the experiments cannot be excluded, a high degree of niche overlap has been recorded in co-occurring Dryopoidea species (Tavares & Williams, 1990; Martins & Ferreira, 2020) and such interactions seem highly unlikely and are not supported by the results of the beetle presence experiment below, or by observations in the Comal Springs system, where there can be dozens of individuals of three species of Elmidae and *S*. *comalensis* on small pieces of wood (∼30 cm^2^). Both species were collected from the Comal Springs system during 2023–2024 for use in these experiments. Work with *S*. *comalensis* was conducted under permits #ES037155 from the US Fish and Wildlife Service and #SPR-0101-131 from the Texas Parks and Wildlife Department.

**Figure 1:**
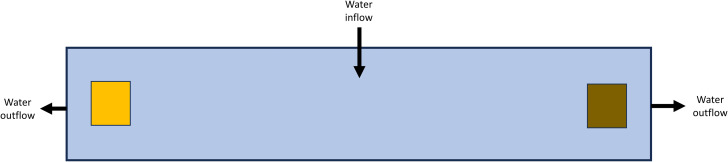
General experimental chamber layout for paired habitat choice experiments. Illustration of the layout of the primary design for paired habitat choice experiments. Figure is from the top-down perspective on a 30 cm × 7.5 cm × 7.5 cm chamber with water inflow at center and outflow at both ends. Colored squares represent two different objects on opposite side of the chamber (*e.g.*, wood and rocks, *etc.*). Figure is not to scale.

All except one of the experiments were paired habitat choice experiments wherein beetles were held in an experimental chamber with one environmental condition at one side of the chamber and a different environmental condition at the opposite end (Fig. 1). After testing some initial designs, an experimental chamber was created out of an elongate plastic container (30 cm long × 7.5 cm wide × 7.5 cm tall). The design of these chambers had some slight modification depending on the purpose of the experiment and is noted for each study below. Generally, all sides of this chamber (including the lid) were blacked out to prevent any light from entering the chamber. Outflow holes were cut into each end of the chamber 1.5 cm below the chamber top and covered with fine mesh such that a filled chamber held ∼1,350 mL of water. The chambers were set up with a flow-through system using water from the Edwards Aquifer: water entered *via* a tube at the top center of each chamber at a rate of approximately 1.5 mL/s, flowed towards each end, and then out each side and was not recirculated (Fig. 1). The entirety of each experimental setup was covered in 6 mil (0.15 mm thick) black plastic to eliminate the influence of other light sources; with the exception of the light experiment, no light was provided to the experimental chambers. The experiments were maintained indoors with climate-controlled conditions at the San Marcos Aquatic Resources Center, San Marcos, Texas.

In each experiment, as many aspects of the setup were randomized as possible, including treatment positions, beetle assignment to experimental chamber, chamber order, *etc.* The experimental chambers and most materials placed within the chambers were cleaned and dried between experiments to remove any debris, environmental cues, or any other buildup within the chambers; materials that were not cleaned (leaves, wood) were discarded between experiments. At most one individual of each species/life stage was placed in each experimental chamber so that individuals could be tracked across days of the experiment. The rarity of *S*. *comalensis*, collection limitations, and long-term survival (no beetles died during experiments, but mortality did occur during intervening periods) restricted the ability to achieve higher levels of replication (but replication was sufficiently powerful here to assess many of the comparisons) or utilize alternative experimental designs with multiple beetles of the same species per chamber (*e.g.*, Cooke, Longley & Gibson, 2015). Here, replication is at the level of individual beetles, and I aimed for a minimum replication of eight individuals of each species for each experiment, but this number varied by study based on the number of beetles available at the time of the experiment. Multiple temporal rounds of the experiments were performed with new beetles if necessary to achieve greater replication (*i.e.,* experiments were repeated at later dates when completely new sets of beetles were available in order to achieve higher replication). Rarely, beetles were not observed during daily checks, likely due to being wedged in interstitial spaces of monitored materials, which I did not want to deconstruct until the experiment ended; usually, the unobserved beetles were seen the following day.

In most of the experiments, the response variable was the position of the beetle within the experimental chamber (*e.g.*, light side or dark side of chamber; categorical variable). Therefore, most analyses are tests of differences in overall proportions between species or among experiments; exact methods are described for each study. Data analysis was performed in R v. 4.4.1 (R Core Team, 2025) using the lme4 v. 1.1-35.5 and lmerTest v. 3.1-3 packages (Bates et al., 2015; Kuznetsova, Brockhoff & Christensen, 2017). Except as noted below, data were analyzed using mixed effects logistic regressions with the ‘glmer’ function fit with a binomial distribution (link = ‘logit’). One additional experiment that attempted to investigate feed rates of larvae on leaves of different tree species is presented in the supplements. Voucher specimens of adults were deposited at the University of Texas Insect Collection, Austin, Texas, and data are available in Figshare (Pintar, 2025).

### Responses to light

Although *S. comalensis* have greatly reduced visual organs (Barr & Spangler, 1992), no direct responses of this species to different light wavelengths have been tested. A basic understanding of responses to light can inform us as to why this species occurs in certain habitats and what conditions refugia populations should be kept in, as well as to inform how the influence of external light is handled in other experiments. To accomplish this, six separate and consecutively conducted experiments were performed to test the response of beetles to different wavelengths of light: a control (no light), white, red, green, blue, and UV light. The UV light was produced by 9W LED UVA 395–400 nm bulbs. The other bulbs were Great Value 9W LED bulbs (red, green, blue, and daylight).

For each experiment, one end (7.5 cm × 7.5 cm) of the experimental chamber and a 7.5 cm × 5 cm section of the lid at the same end were not blacked out so that light could enter the chamber. Lightbulbs were placed outside of the chamber but underneath the black plastic sheeting and set with a timer to a 12:12 h light:dark cycle from 06:00 to 18:00. Two bulbs were hung approximately 50 cm apart and 20 cm above one end of the experimental chambers; all experimental chambers had the treatment (open light end) on the same side for this experiment. One piece of wood (∼3 cm × 1.5 cm × 0.5 cm) was placed at each end of the chamber to serve as a food source and substrate; each piece of wood was placed below a small limestone rock (∼1.5 cm diameter) to prevent movement. Beetles were placed in the middle of the chamber and checked between 09:00 and 11:00 the following two or three days (limited by access to the facility). The location of the beetle in the chamber was recorded, as well as the position of the beetle relative to the habitat (on top of the wood/rock or below the wood/rock) each day. Only one light type was tested at a time, so overall replication varied between tests; total numbers of beetles by species and experiment are listed in Table 1.

**Table 1:** Responses of beetles to light types by side of the experimental chamber. Responses of beetles to light types. N indicates number of unique beetles tested for each species/life stage. The number of total observations (counting the same beetle across multiple days in each experiment) beetles were observed on the dark and light sides the experimental chamber when the light side was illuminated by white, blue, green, red, or UV light and the control (no light). Mixed effects logistic regression results are in reference to white light and coefficient indicates direction and magnitude of response relative to white light with negative values indicating higher proportions in darkness. Bold indicates significant differences (*P* < 0.05) from white light.

	N	Dark	Light	% Dark	Coef.	*z*	*P*
*Stygoparnus* adults							
White	10	2	18	10.0			
Blue	9	13	14	48.1	−3.84	−2.07	**0.038**
Green	9	15	11	57.7	−4.82	−2.29	**0.022**
Red	9	3	15	16.7	−0.65	−0.37	0.71
UV	10	18	2	90.0	−7.90	−3.15	**0.0017**
No light	10	9	10	47.4	−3.85	−2.02	**0.043**
*Stenelmis* adults							
White	10	17	2	89.5			
Blue	11	26	7	78.8	0.90	0.94	0.35
Green	12	22	12	64.7	1.66	1.79	0.074
Red	9	8	9	47.1	2.48	2.43	**0.015**
UV	12	12	12	50.0	2.36	2.43	**0.015**
No light	12	11	13	45.8	2.55	2.60	**0.0093**
*Stenelmis* larvae							
White	10	15	4	78.9			
Blue	11	17	11	60.7	1.05	1.32	0.19
Green	13	26	13	66.7	0.79	1.04	0.30
Red	9	12	6	75.0	0.84	0.82	0.41
UV	11	13	8	61.9	0.93	1.15	0.25
No light	12	11	13	45.8	1.66	2.07	**0.039**

Data from each of the six separate light experiments were combined for analysis. I conducted two mixed effects logistic regressions with a binomial distribution on each species/life stage. Although some beetles were reused in subsequent experiments, due to communal housing between experiments, individuals could not be tracked between the studies. However, because individuals were tracked between consecutive days of the same experiment, I included experimental container and day (day 1 or 2 of the experiment) as random effects to account for container or day-specific effects, respectively. In the first analysis, the proportion of beetles on each side of the experimental chamber (dark or light) was the response variable and light type was the predictor variable. In the second analysis, the position of the beetle relative to the wood structure—hidden under the wood or in the open exposed to light (beetles ‘on wood’ and beetles ‘walking in the open on the bottom of the container’ were combined for analysis purposes)—was the response variable with light type as the predictor variable. The initial experiment used white light and served as the baseline from which all other light types were compared in both analyses. Use of white light as a baseline is necessary from a statistical perspective to have a point from which to compare other proportions but also represent a broader spectrum of light encompassing the other visible wavelengths (excluding UV).

### Physical habitat structure

Given the reported association of *S*. *comalensis* with sycamores (BIO-WEST, 2022), experiments were performed to determine whether *S. comalensis* has a preference for, or attraction towards, wood. Two paired habitat experiments were conducted to test the relative preference of beetles for conditioned dead sycamore wood relative to two other objects: leaves and rocks. The wood was conditioned for approximately one year (approximate duration after which wood is well-conditioned and sinks) and leaved for one month using a flow-through system with water from the Edwards Aquifer. The initial experiment paired a limestone rock and a similarly sized piece of sycamore wood (∼3 cm × 1.5 cm × 0.5 cm), while the second experiment paired sycamore wood with a similarly sized clump of sycamore leaves. In the second experiment, a limestone rock was placed on top of the leaves to hold them in place and also on top of the wood to maintain a symmetrical design. Beetles were set in the middle of the experimental chamber and checked after 24 h when the side of the experimental chamber and the beetle position (on top of or below wood/rock/leaf) were recorded. After the initial check, beetles were reset by placing them in the middle of the chamber and then checking after another 24 h before ending the experiment. There were 12–24 replicates for each species/life stage and habitat pair.

Data from the two paired habitat experiments were analyzed individually (by experiment) and together (both experiments combined) to compare responses among and within species, respectively. I used mixed effects logistic regressions with a binomial distribution for all analyses. Similar to the light experiment, some individuals were reused in subsequent experiments (not tracked between experiments) but were tracked between consecutive days of the same experiment; experimental container and day were included as random effects. First, I tested whether the proportion of beetles on the wood side of the experimental chamber varied in *S*. *sexlineata* adults and larvae relative to *S*. *comalensis* adults for both the wood *vs.* rock and wood *vs.* leaf experiments separately. Second, I analyzed whether the proportion of individuals above the wood/rock/leaf varied among species in the two experiments separately. Then, I combined the data from both experiments and tested for differences in proportions within species between the two experiments; this analysis was to determine if the relative use of wood as a habitat changed when the alternative was a rock *versus* a leaf.

### Beetle presence

This experiment assessed whether adult *S*. *comalensis* and adult *S*. *sexlineata* respond to the presence of caged conspecifics and heterospecifics. The experimental design utilized one caged adult beetle and one free-roaming adult beetle within the same experimental chamber. Cages were constructed out of 50 mL polypropylene tubes (25 mm diameter) with screw caps (Fig. S1). The tubes were cut to a length of 40 mm, fine mesh was hot-glued to cover the open end of the tube, and four 3-mm diameter holes were drilled into the cap and covered with fine mesh. This allowed movement of water and dispersion of cues between the cage and the rest of the experimental container. Cages contained one piece of wood (∼3 cm × 1.5 cm × 0.5 cm) to serve as a food source and one small limestone rock (∼1.5 cm diameter) to prevent the cage from floating. One cage was placed at each end of the experimental chamber for a symmetrical design; the side that received the beetle was randomly selected. One similar-sized piece of wood and rock were placed next to each cage (outside of the cage) in the experimental chamber to serve as food and additional substrate for the free-roaming beetle. At the start of the experiment, the free-roaming beetle was placed in the center of the experimental chamber. The location of the free roaming beetle was recorded once per day for the following three days before each replicate was terminated. After checking and recording the position of each beetle, its position was reset by placing the beetle at the center of the experimental chamber. All *S*. *comalensis* in this experiment were females (determined following Kosnicki, 2019); *S*. *sexlineata* are not identifiable to sex in the same manner and therefore were not sexed.

Six separate temporal rounds of the experiment were conducted to achieve at least eight replicates of each of the four species pairs: responding *S*. *comalensis* and caged *S. comalensis* (*N* = 14 beetles), responding *S*. *comalensis* and caged *S*. *sexlineata* (*N* = 11), responding *S*. *sexlineata* and caged *S*. *comalensis* (*N* = 8), and responding *S*. *sexlineata* and caged *S. sexlineata* (*N* = 16). Individual beetles only acted as the responding beetle or caged beetle at most once for each of the species pairs. I used mixed effects logistic regressions with a binomial distribution for two analyses. For each species, I separately tested whether the proportion of beetles adjacent to the cage containing a beetle was different between heterospecifics and conspecifics. Day and experimental chamber were again included as random effects in these analyses.

### Wood conditioning

I conducted two experiments to test beetle responses to captive- *versus* wild-conditioned sycamore wood. Wood conditioned in captivity could have different microbial communities (Mays et al., 2021) that make it more palatable to beetles (Danger et al., 2012), which could in turn potentially affect beetle fitness. The captive wood was conditioned using a flow through system with their aquifer water supply for over one year (part of a conditioning system that pre-dated this study). The wild wood (bark-free) was collected from the Spring Island area of the Comal Springs system the morning that the experiment started and had been in the water for an unknown amount of time. Wild wood of similar general integrity to the captive conditioned wood (based on course touch) was selected; the long time (∼1 year) necessary for controlled conditioning of wood in both the wild and captivity was not feasible given the timeline of these studies. The wild-collected wood was checked in the field and then in the lab under a microscope to remove any obvious invertebrates.

The first experiment was a selection experiment in which one piece of wood (∼3 cm × 1 cm × 0.5 cm) was placed at each end of the experimental chamber. One *S*. *comalensis* adult and one *S*. *sexlineata* adult were randomly assigned to each experimental chamber (*N* = 12 beetles for each species) and placed in the center and checked once per day on the following three days. After each daily check, the beetles were reset by placing them at the center of the chamber. Differences in proportions of beetles on each type of wood (response variable) between the two species (predictor variable) were analyzed using a mixed effect logistic regression with a binomial distribution that included day and experimental chamber as random effects.

The second experiment investigated whether feeding rates by *S*. *comalensis* differed between the captive and wild-conditioned wood. Because of their translucent cuticles, we are able to see material within the abdomen of *S*. *comalensis* when backlit (similar to Kosnicki, 2019; Fig. S2); *S*. *sexlineata* are too dark for this method to work. After beetles were photographed, I quantified the proportion of the abdomen below the elytra that was full of gut contents by measuring the areas using ImageJ (Schneider, Rasband & Eliceiri, 2012). This experiment consisted of two rounds with eight individual beetles that were tracked and repeated between rounds. Each experimental chamber received one piece of wood (∼3 cm × 1 cm × 0.5 cm) and beetles were randomly assigned to experimental chambers during both rounds. During the first round, half of the chambers were randomly assigned to receive captive wood and the other half received wild wood; during the second round the wood treatment was alternated.

On day 0 of the experiment (setup day), beetles were placed in the center of the experimental chamber with one limestone rock (∼1.5 cm diameter) placed in the center of the chamber. No wood was initially placed in the chamber so that I could determine how much of their guts is cleared in one day. In all cases, beetles showed no signs of food in their guts after only 24 without a food source (Fig. S2). After 24 h (on day 1), the wood was added to the center of the chamber and the rock was removed. After another 24 h (on day 2) the wood was removed, beetles were photographed, and the rock was replaced. I repeated this process once more to obtain two measurements of gut contents, on days 2 and 4 of each round of the experiment. The proportion of the abdomen area occupied by gut contents (response variable) was analyzed using mixed effects models with ‘lmer’ function. Wood conditioning was the fixed predictor variable, and beetle individual, round of the experiment, and day of each trial (2 or 4) were random effects.

### Response to flow

As a spring-endemic species, we might expect *S. comalensis* to have an affinity for flowing water. I investigated whether *S*. *comalensis* occupied habitat within the experimental chambers based on where there was water flow. In all of the above studies, the water inflow tube was hanging above the center of the experimental chamber, inaccessible to any beetles. Additionally, results of the above experiments indicated that *S*. *comalensis* have a strong attraction to wood and preference for clinging to structures within the experimental chambers. In this study, the outflow at one end of the experimental chamber was blocked off such that water would only flow out the opposite side (Fig. S3). At each end of the chamber, a piece of wood was placed vertically that stretched from the bottom of the experimental chamber to the outflow (one cm below the top).

I then varied where the inflow tubing was placed through three iterations of the experiment. First (Fig. S3A), I placed it in the center just dangling into the top of the water as in the previous studies, with one end of the chamber having outflow and the other effectively no flow. Second (Fig. S3B), I placed the inflow adjacent to the piece of wood at the end of the chamber opposite of the outflow, creating unidirectional flow across the chamber. Lastly (Fig. S3C), I placed the flow directly adjacent to the piece of wood at the outflow, largely limiting any flow across the chamber. Beetles were placed in the center of the chamber and checked after 24 and 48 h. After the first check, beetles were reset and placed in the center of the chamber. Only one beetle was placed in each experimental chamber and some beetles were repeated between the three iterations of the experiment; these individuals were tracked between iterations (*N* = 8 individual beetles).

I analyzed the data twice with mixed effects logistic regressions with binomial distributions. First, I tested whether the proportion of beetles on the outflow varied between the three setups. Second, I tested whether the proportion of beetles at the location of strongest accessible flow varied between the three setups. I considered the strongest flow to be at the outflow in Fig. S3A because the inflow point was inaccessible. In Fig. S3B, the strongest flow was at the inflow because it is concentrated at the point of the small inflow tubing. In Fig. S3C, inflow and outflow were at the same side, so this was also the point of strongest flow.

## Results

### Responses to light

Results of the light experiments indicated there was significant variation in the position of *S*. *comalensis* adults, *S*. *sexlineata* adults, and *S*. *sexlineata* larvae in response to different light types (Table 1). *Stygoparnus comalensis* adults were attracted to white and red light, with only 10% and 17% of recorded locations, respectively, being on the dark side of the experimental container. Significantly fewer *S*. *comalensis* were recorded on the light side for all other light types: approximately 50% (no preference between light and dark) were observed on each side with blue, green, and no light (control). Ninety percent of *S*. *comalensis* were on the dark side in UV light, indicating avoidance of UV light. *Stenelmis sexlineata* adults selected the dark side of the experimental chamber when white, blue, and green light were present but showed no response to red, UV, or no light. *Stenelmis sexlineata* larvae were found on the dark side of the experimental chamber in the presence of all light types, but when no light was present, they were found on both sides approximately equally (46% dark).

*Stygoparnus comalensis* adults showed no variation in their microhabitat use (above or below wood) in response light type (Table 2); overall, 71% of *S*. *comalensis* were on top of the wood or otherwise exposed to light. *Stenelmis sexlineata* adults showed a similar pattern of microhabitat use as they did when selecting the side of the experimental chamber: when in the presence of white, blue, or green light >90% of *S*. *sexlineata* adults were found under wood with no variation among light types. *Stenelmis sexlineata* larvae were universally found under wood when any light type was present, with only 2 out of 24 occurrences in the open when no light was present.

**Table 2:** Responses of beetles to light types by position within the experimental chamber. Responses of beetles to light types: number of total observations (counting the same beetle across multiple days in each experiment) beetles were observed under wood, on top of wood, or crawling in the open when the light side was illuminated by white, blue, green, red, or UV light and the control (no light). Mixed effects logistic regression results are in reference to white light and coefficients indicate direction and magnitude of the response with a higher proportion under wood (negative) or in the open exposed to light (positive) relative to the response to white light. *Stenelmis* larvae were not analyzed due to no variation across five of the six treatments.

	Under	On	Open	% Under	Coef.	*z*	*P*
*Stygoparnus* adults							
White	6	13	1	30			
Blue	16	10	1	59	1.22	1.95	0.051
Green	5	20	1	19	−0.59	−0.84	0.40
Red	2	15	1	11	−1.23	−1.38	0.17
UV	2	16	2	10	−1.35	−1.52	0.13
No light	7	10	2	37	0.31	0.45	0.65
*Stenelmis* adults							
White	19	0	0	100			
Blue	30	3	0	91	0.35	0.01	0.92
Green	31	3	0	91	0.31	0.01	0.93
Red	13	4	0	76	−0.12	−0.0	0.97
UV	18	4	0	82	−0.49	−0.1	0.97
No light	16	8	0	67	−0.88	−0.3	0.78
*Stenelmis* larvae							
White	19	0	0	100			
Blue	28	0	0	100			
Green	39	0	0	100			
Red	18	0	0	100			
UV	21	0	0	100			
No light	22	0	2	92			

### Physical habitat structure

*Stygoparnus comalensis* adults were found on wood at high rates in both experiments: 96% of observations were on wood when rock was the alternative and 79% when leaf was the alternative (Table 3). These were significantly higher rates than either *S*. *sexlineata* larvae or adults in both experiments. The occurrence of *S*. *comalensis* on wood when rock was the alternative was significantly higher than when leaf was the alternative (Tables 3, 4). Both *S*. *sexlineata* larvae and adults were found on/under wood >50% of the time when rock was the alternative. However, while use of wood did not significantly vary between experiments for *S*. *sexlineata* adults, there was a large shift toward favoring leaves among *S. sexlineata* larvae when offered the option (Table 4).

**Table 3:** Results of the two paired physical habitat structure experiments. Results of two paired habitat experiments, wood *vs.* rock and wood *vs.* leaf. N indicates number of unique beetles tested for each species/life stage. The number of total observations (counting the same beetle across multiple days in each experiment) beetles were observed on the “wood” or on the “other” object (rock or leaf) when comparing Side and the percent of observations the beetle was above the object when comparing Position. Mixed effects logistic regression results are in reference to adult *S. comalensis*. Bold indicates significant differences (*P* < 0.05).

	N	Wood	Other	%	Coef.	*z*	*P*
Side							
Wood *vs.* Rock							
*Stygoparnus* adults	24	46	2	96			
*Stenelmis* adults	20	25	15	63	−2.62	−3.31	**0.0009**
*Stenelmis* larvae	14	22	6	79	−1.84	−2.14	**0.032**
Wood *vs.* Leaf							
*Stygoparnus* adults	19	30	8	79			
*Stenelmis* adults	16	15	17	47	−1.73	−2.69	**0.0072**
*Stenelmis* larvae	12	3	21	13	−3.69	−4.13	**<0.0001**
Position							
Wood *vs.* Rock							
*Stygoparnus* adults	24	30	18	63			
*Stenelmis* adults	20	10	30	25	3.69	3.02	**0.0025**
*Stenelmis* larvae	14	1	27	4	7.97	3.08	**0.0021**
Wood *vs.* Leaf							
*Stygoparnus* adults	19	22	16	58			
*Stenelmis* adults	16	16	16	50	0.33	0.66	0.51
*Stenelmis* larvae	12	2	22	8	2.74	3.33	**0.0009**

**Table 4:** Comparisons of responses between experiments for the pair physical habitat structure experiments. Results of comparisons between experiments within each species of the side and position of beetles. For side, each row indicates whether the proportion of beetles on wood was lower (significant positive coefficient) when wood was paired with a leaf in reference to when wood was paired with a rock. For position, each row indicates whether the proportion of beetles above the object was higher (positive coefficient) or lower (negative coefficient) when wood was paired with a leaf in reference to when wood was paired with a rock. Results are those from mixed effects logistic regression. Bold indicates significant differences (*P* < 0.05).

	Coef.	*z*	*P*
Side paired with leaf, *vs.* rock			
*Stygoparnus* adults	1.87	2.09	**0.036**
*Stenelmis* adults	0.63	1.17	0.24
*Stenelmis* larvae	3.16	3.98	**<0.0001**
Position paired with leaf, *vs.* rock			
*Stygoparnus* adults	0.28	0.43	0.67
*Stenelmis* adults	−1.25	−1.92	0.055
*Stenelmis* larvae	−0.90	−0.71	0.48

The position that *S*. *comalensis* adults were found in was “on top of the object” for 63% of the observations for wood *vs.* rock and 58% of the time for wood *vs.* leaf, significantly greater than either *S*. *sexlineata* adults or larvae (Table 3). *Stenelmis sexlineata* were typically found underneath the wood, rocks, or leaves, or in the case of larvae, typically within the folds of the leaves. While the position of each taxon did not significantly vary between the two experiments, there was a marginally higher occurrence of *S*. *sexlineata* adults above the object when leaf was the alternative than when rock was the alternative (Table 4).

### Beetle presence

A majority of free-roaming *S*. *comalensis* (77%) and *S*. *sexlineata* (68%) were adjacent to the cage when conspecifics were in the cage, but approximately half of occurrences were next to the cage when heterospecifics were in the cage (50% *S*. *comalensis*; 46% *S*. *sexlineata*). This was a significantly higher proportion of beetles next to conspecifics *versus* heterospecifics for *S*. *comalensis* (*z* = 2.20, *P* = 0.027), but this was a marginally non-significant difference for *S*. *sexlineata* (*z* = −1.67, *P* = 0.095). While all beetle positions (object they were crawling on) were not recorded in this experiment, *S. comalensis* were observed crawling on the mesh side of the cage when another *S. comalensis* was in the cage, in contrast to typical positions for other species pairings (*S. comalensis* on wood and *S. sexlineata* under wood).

### Wood conditioning

The number of beetles on captive *versus* wild wood was approximately even and not significantly different between species (*z* = −0.23, *P* = 0.82). There were 20 occurrences of *S*. *comalensis* on captive wood and 19 on wild wood, whereas there were 21 occurrences of *S*. *sexlineata* on captive wood and 18 on wild wood.

**Figure 2:**
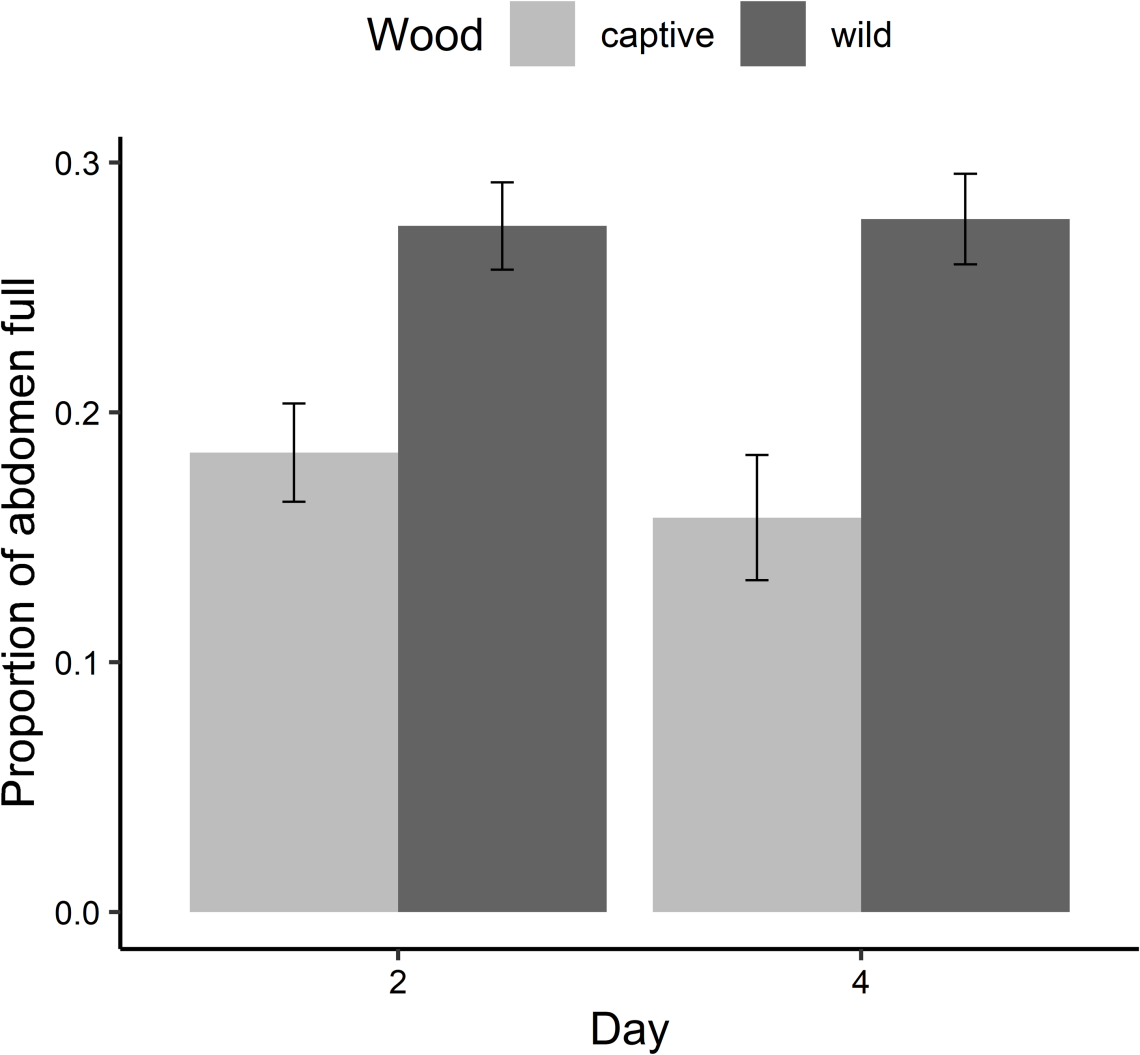
Results of the conditioned wood feeding experiment. The proportion of the abdomen (mean ± SE) of *S*. *comalensis* adults occupied by gut contents by day of the experiment. Proportion was determined from dorsal backlit photos when beetles were provided with captive or wild conditioned sycamore wood. Days 1 and 3 were following gut clearing and beetles had no detectable gut contents.

*Stygoparnus comalensis* with wild-conditioned wood had significantly more of their abdomen full of gut contents (Fig. 2; 27.6 ± 1.2%; mean ± standard error (SE)) than those with captive-conditioned wood (17.1 ± 2.6%) (*F*_1,30_ = 27.9, *P* < 0.0001). The proportion of guts full did not vary between days 2 or 4 or between rounds of the experiment.

### Response to flow

There were no significant differences in where adult *S*. *comalensis* were observed between the three setups (Tables S1, S2), but there was a marginal difference in the proportion on the outflow between when the inflow was at the opposite end and when it was adjacent to the outflow. This indicates there may be some potential affinity for flow, but it is not as strong as with the affinities of *S*. *comalensis* for some other habitat conditions such as wood and light. In this experiment, beetles were always observed on the wood, but there was no consistent position in the water column where they occurred. Although the wood extended slightly above the water surface, no beetles were ever observed above the water surface.

## Discussion

*Stygoparnus comalensis* clearly demonstrated a strong affinity for wood in these experiments—a habitat preference that was not consistently observed in the co-occurring *S*. *sexlineata*. The physical habitat structure experiment directly tested this and showed a strong preference by *S*. *comalensis* for wood over leaves or rocks. Because results of the wood conditioning experiment clearly show they are feeding on wood and/or their biofilms, a behavior and association reported among related species (Phillips, 1995; McKie & Cranston, 1998; Hoffmann & Hering, 2000; Eggert et al., 2012), their habitat preference for wood is likely a response to some sort of chemical cue produced by the wood directly or by biofilms that may be decomposing the wood (Jonsell & Nordlander, 1995), as even aquatic insects with developed eyes rely on chemotaxy for locating resources (Brönmark & Hansson, 2000; Bilton, Freeland & Okamura, 2001; Röder, Mota & Turlings, 2017). In contrast, *S*. *sexlineata* adults had only slightly more (63%) occurrences on wood *versus* rock and relatively even occurrences on wood (47%) *versus* leaves. However, anecdotal observations from experimental and holding chambers indicate that in the absence of wood, *S. comalensis* are seemingly both more active (observed crawling across the chamber) and have an affinity for clinging to some sort of object (*i.e.,* not the smooth side of the chamber). These other occurrences were not tracked consistently and so are not presented here, but further suggest that *S*. *comalensis* actively seeks out wood.

Biofilms that grow on wood serve as a food resource for *S*. *comalensis* (Nair, Diaz & Nowlin, 2021) and are seemingly the preferred food source over biofilms that may be growing on leaves. This is in contrast to *S. sexlineata* larvae, which were predominantly found within or under leaf packs when paired with wood; whether this is a true preference for leaves as a food source or a preference for the greater interstitial space they provide (between the many crevices in a folded leave *versus* only underneath the piece of wood) could not be discerned here, but substrate size is an important determinant of the occurrence of some elmid species (Yoshimura, 2012; Fujiwara, Maeto & Yoshimura, 2022). In the wood conditioning study, adult *S. comalensis* consumed more when paired with wild-conditioned wood *versus* wood conditioned in captivity. Whether this is a difference in the quality or quantity of wood/biofilms is unknown, but beetles were provided with equal sized pieces of wood from each source, so the amount of surface area for biofilm to grow on each piece should have been relatively similar. Regardless, results of both of these studies indicate that *S. comalensis* maintained in captivity should be provided with wood, and the relative amount of captive-conditioned wood needed to sustain a population could be higher than would be provided from wood collected in the wild. Furthermore, lower consumption rates of captive-conditioned wood perhaps suggest there are issues with the quality of biofilm on captive-conditioned wood; microbial communities are known to be different between captive and wild beetles in the Comal Springs system (Mays et al., 2021), but any implications of these differences are unknown. Biofilm quality could have consequences for successfully maintaining captive populations and be one of the reasons behind why there has been limited success breeding this species and raising larvae to the adult stage in captivity.

There was no clear response of *S. comalensis* to flow in the flow experiment presented here or in preliminary tests of responses to flow. Despite being a species that only occurs in springs, this lack of response could be due more to the limitations of the experimental design rather than any true lack of affinity for flowing water. The small experimental chamber may not have provided sufficient variation in flow or associated differences in water quality that would elicit a response by *S. comalensis*. Additionally, most records of *S*. *comalensis* are from low-volume springs (Barr, 1993), so it possibly prefers lower-flow springs, but responses across a wider gradient of flows remain unknown. The affinity for wood may override any other factors with minor variation, such as flow, in these experiments.

The tendency of free-roaming *S. comalensis* to occupy habitats closer to conspecifics but not heterospecifics suggests an attraction to beetles of the same species; a similar but non-significant tendency was observed with *S*. *sexlineata*. In aquatic systems and among insects more broadly, semiochemicals (information-carrying chemicals) are widely used by animals to inform them of environmental conditions and select habitats (Brönmark & Hansson, 2000; Crespo, 2011; Eveland et al., 2016), including attraction towards, and avoidance of, other aquatic beetles (Pintar & Resetarits, 2020). Therefore, it should especially be expected that a species occupying dark habitats and lacking developed eyes would rely on chemicals produced by conspecifics for finding mates or to find favorable habitats that are already occupied by others of the species (Buxton et al., 2020). This is further supported by initial field studies (BIO-WEST, 2025, unpublished data) in which 57% of samples where *S. comalensis* were found (representing 82% of beetles), more than one individual found.

Both beetle species here displayed phototaxis (movement in response to light). Whereas *S*. *sexlineata* adults avoided three light types (white, blue, and green) and did not respond to two types (red, UV), perhaps the most peculiar of all the experimental results are the responses of *S. comalensis* to light: they avoided UV light, had no response to blue or green light, but were attracted to white and red light. The positive response to white light could just be a response to the red component of light emitted by those lights. The opposite responses to UV and red light also coincide with the wavelengths on opposite sides of the spectrum. Different insect species respond to different wavelengths of light in various ways (Menzel & Blakers, 1976; Peitsch et al., 1992; Koshitaka et al., 2008; Kühne et al., 2021), but attraction to red or infrared light appears to be somewhat common (Park & Lee, 2017). Why *S. comalensis* seems to respond to this wavelength is unknown but is perhaps an adaptation that has not been lost with the reduction of its visual organs; some aquatic beetles with reduced visual organs maintain some sensitivity to light (Langille et al., 2019). However, whether there might ever be sufficient light of these wavelengths in the absence of UV light to affect behavior of *S*. *comalensis* is unknown. Similarly, responses to UV light are common in insects (usually attraction; Fraleigh, Heitmann & Robertson, 2021), but the avoidance of UV light by *S. comalensis* at least aligns with its occupancy of subsurface habitats. Detection of UV light during daylight hours would be an indication that a beetle has strayed too close to the spring surface and away from favorable habitats. This could be useful for a species that seems to have little response to flow, as well as specifically for the environmental conditions in Comal Springs where there is relatively little variation in water quality that beetles could respond to across the small distances near spring openings. In captive populations, *S*. *comalensis* could be kept in conditions with diurnal light exposure (as long as some dark refuges are also included), but whether light could affect other aspects of its biology remains unknown. Exposure to any light source or removal of natural light regimes could potentially elicit responses or induce stress in the beetles that could affect behavior and reproduction (Owens & Lewis, 2018; Desouhant et al., 2019).

In contrast to the responses by *S*. *comalensis* to light, the responses by *S. sexlineata* to light were not surprising: larvae nearly completely avoided all light while adults avoided white, blue, and green light. These experimental observations are consistent with field observations. When checking springs during the daylight hours, both adults and larvae of *S. sexlineata* have always been seen in darker conditions, such as on the underside of wood in springs; similar light avoidance is seen in the Comal Springs system by *H*. *comalensis* (Cooke, Longley & Gibson, 2015). Furthermore, other species of *Stenelmis* have been documented to be more active at night (Cowell & Carew, 1976). Larvae also have a strong affinity for occupying interstitial space within leaves or under wood, so these two aspects could not necessarily be separated with the results here, but we would expect larvae to avoid light to at least a similar degree as the adults do. Much more study would be required to fully understand the behavioral and physiological basis behind the responses to light in both *S*. *sexlineata* and *S*. *comalensis*.

## Conclusions

This series of controlled habitat choice experiments have tested and confirmed some of the previously stated but untested ideas about the ecology of *S. comalensis*, most notably the attraction to wood, and the responses by *S*. *comalensis* in these experiments in most cases contrast with those of the widespread species *S*. *sexlineata*. These initial findings have since been used to develop preliminary methods for sampling and monitoring wild populations using conditioned pieces of wood and can be used for refining protocols for housing *S. comalensis* in captivity. The suggestions that can clearly be confirmed here—that *S. comalensis* should be housed communally, with conditioned wood as food source, and probably in dark conditions—are not surprising or substantive departures from current protocols. However, some aspects of these findings, particularly the optimal food source (*e.g.*, wild or conditioned wood, different species of wood, etc.), would require further investigation for refining protocols when housing this species in captivity and creating a self-sustaining captive population. The findings here regarding the responses to wood further support the need for not only maintaining healthy tree populations with extensive root networks through the Comal Springs system, but also potentially for maintaining submerged wood within the springs as a food source, as it is not currently known whether live trees are directly important for *S. comalensis* survival and reproduction over short time scales. Much remains unknown about factors affecting *S*. *comalensis* in the wild and captivity, and future research needs to dually approach both to better understand this endangered species.

## Supplemental Information

10.7717/peerj.20507/supp-1 Supplemental Information 1 Supplemental information for ‘Experimental tests of habitat preferences in the Comal Springs dryopid beetle, *Stygoparnus comalensis*’.

## Additional Information and Declarations

### Field Study Permissions

The following information was supplied relating to field study approvals (*i.e.,* approving body and any reference numbers): Work with endangered species was conducted under permits #ES037155 from the U.S. Fish and Wildlife Service and #SPR-0101-131 from the Texas Parks and Wildlife Department.

### Data Availability

The following information was supplied regarding data availability: Data are available at Figshare: - Pintar, Matthew (2025). Data from: Experimental tests of habitat preferences in the Comal Springs dryopid beetle, *Stygoparnus comalensis*. figshare. Dataset. https://doi.org/10.6084/m9.figshare.30350482.v1
