# Supplementary material for: Experimental tests of habitat preferences in the Comal Springs dryopid beetle, Stygoparnus comalensis
Source: PeerJ. 2025 Dec 16;13:e20507. doi: 10.7717/peerj.20507 (PMC12716134; doi:10.7717/peerj.20507)
Supplement: Supplemental Information 1 [file peerj-13-20507-s001.docx]

**Supplemental information for:**

Pintar, MR. Experimental tests of habitat preferences in the Comal Springs dryopid beetle, *Stygoparnus comalensis*. PeerJ. https://doi.org/10.7717/peerj.20507

**Fig. S1.** Photograph of a beetle cage constructed out of polypropylene tubes used for the beetle presence study.


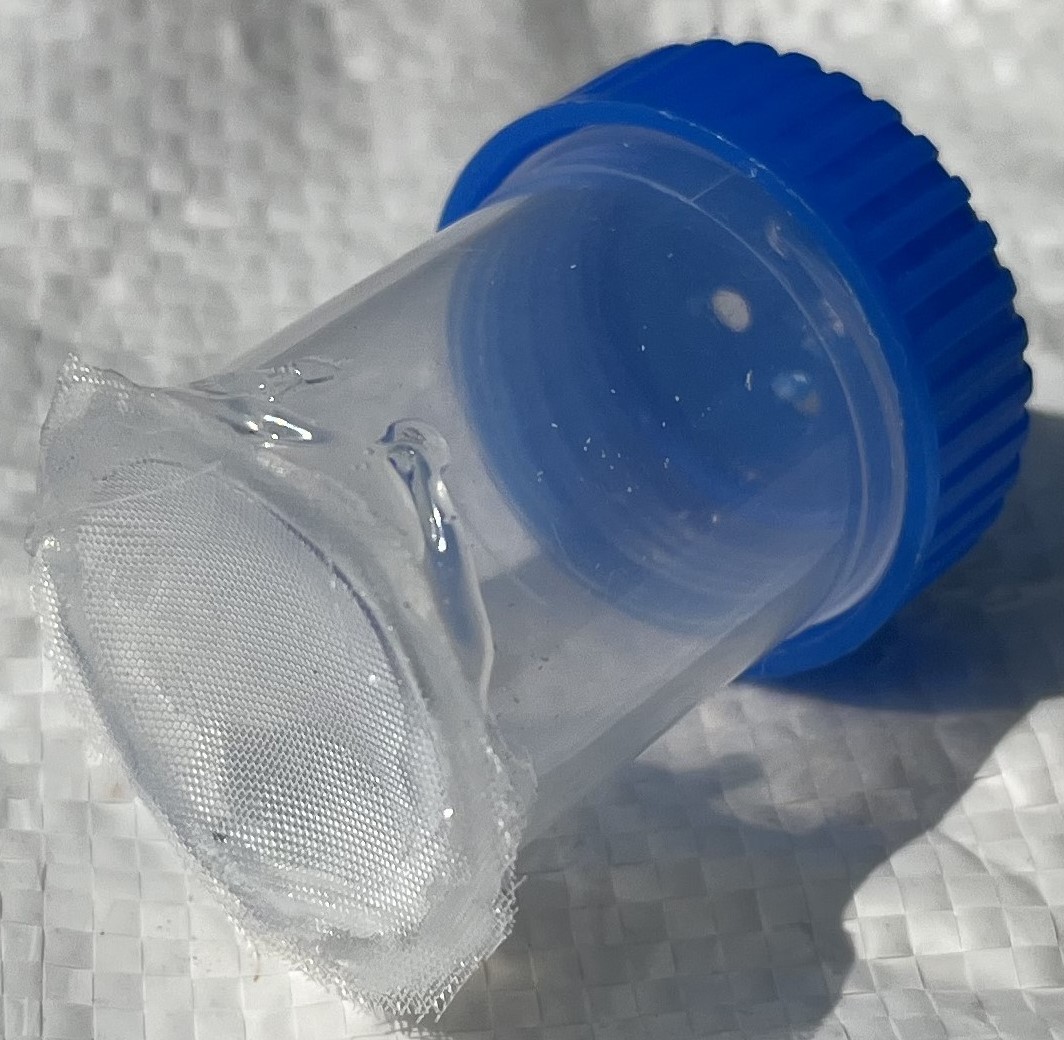


**Fig. S2.** Backlit dorsal photographs of the same beetle on day 0 (start of the experiment), day 1 (after 1 day without food), and day 2 (after 1 day with wild-conditioned wood). The dark areas in the abdomen on days 0 and 2 are gut contents, which are notably absent on day 1.


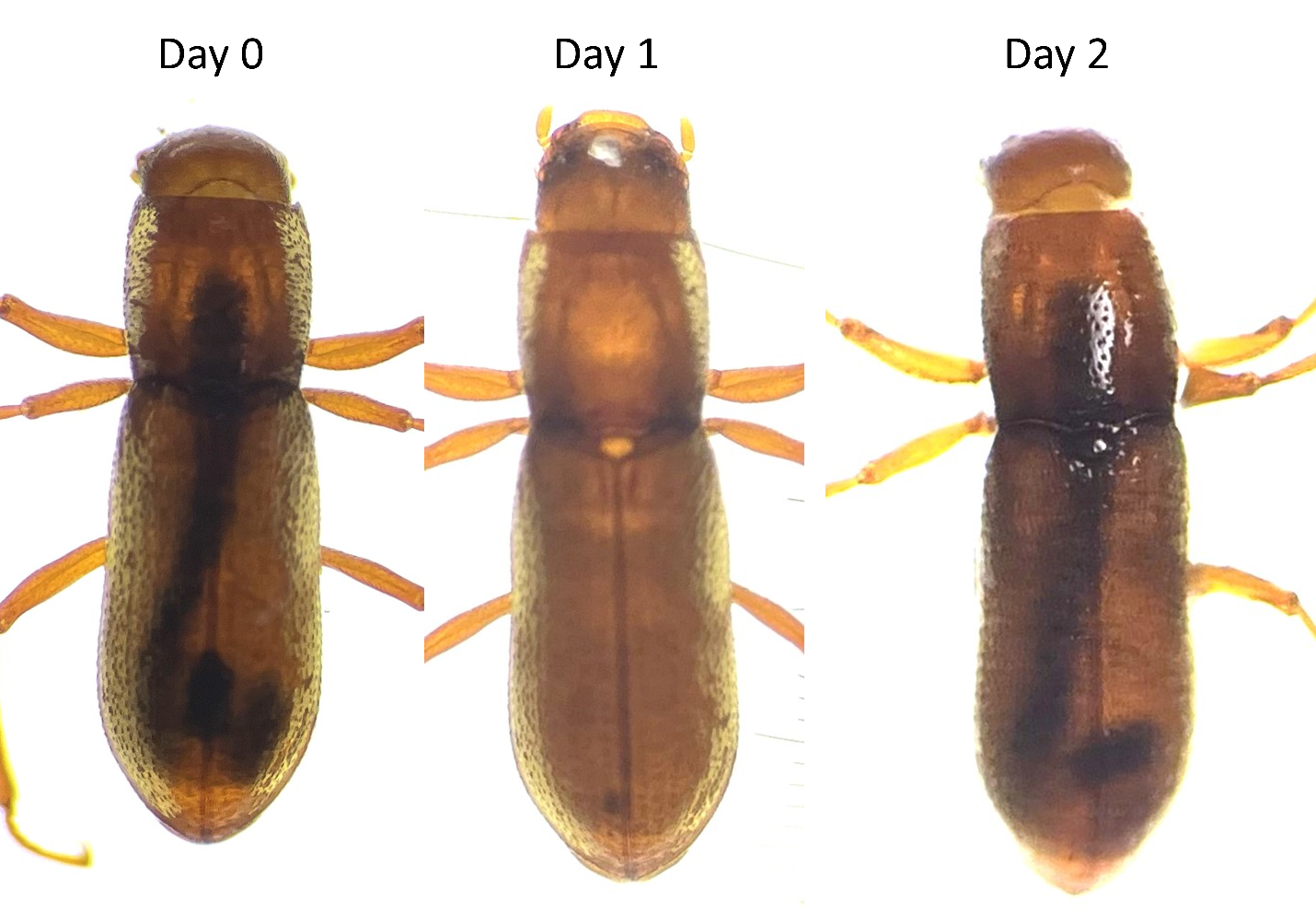


**Fig. S3.** Illustration of the three variations in the experimental setup in the flow experiment. A wood stick was placed vertically at each of the experimental chamber and water was allowed to flow out of the top of one end only. The inflow tube was considered the point of strongest flow (except in A where it dangled at the water surface and was not accessible to beetles). In B and C, the inflow was placed adjacent to the wood. Blue arrows indicate the dominant flow in each setup.


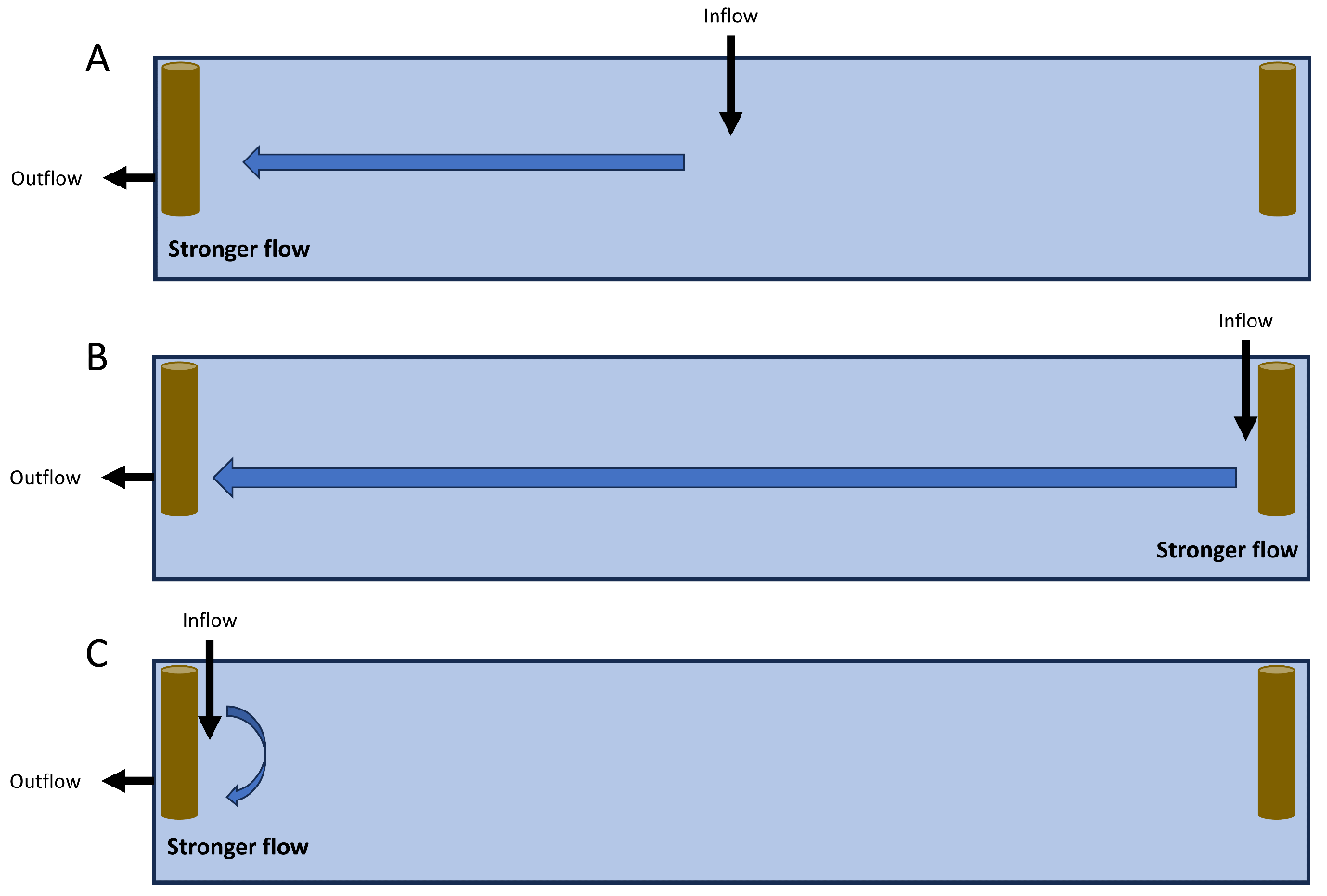


**Table S1.** Number of total observations (counting the same beetle across multiple days in each experiment) of where beetles were observed based on where the inflow was positioned (opposite, middle, or outflow; Fig. S3). The left three columns present the occurrences based on how many beetles were observed on the wood at the outflow versus the opposite end and the percentage of observations on the outflow. The right three columns consider this from the perspective of where the stronger accessible flow was located: this was the inflow when adjacent to a piece of wood (opposite and outflow) or the outflow when the inflow was in the middle.

| Inflow location | Opposite | Outflow | % outflow | Stronger | Lower | % stronger |
| --- | --- | --- | --- | --- | --- | --- |
| Opposite | 16 | 8 | 33 | 16 | 8 | 67 |
| Middle | 11 | 13 | 54 | 11 | 13 | 46 |
| Outflow | 10 | 14 | 58 | 14 | 10 | 58 |

**Table S2.** Results of binomial logistic regressions assessing *S*. *comalensis* responses to flow. The top results are testing whether the proportion of beetles on the outflow side varies based on where the inflow was located (middle or experimental chamber or next to the outflow) and is relative to the inflow being placed at the opposite end of the inflow. The second set of results is assessing whether the proportion of beetles at the location of stronger flow varies relative to when the stronger flow (inflow in this case) is at the opposite end to the outflow.

|  | Coef. | *z* | | *P* | |  |
| --- | --- | --- | --- | --- | --- | --- |
| Proportion on outflow | | |  | |  | |
| Middle | 0.86 | 1.44 | | 0.15 | |  |
| Outflow | 1.03 | 1.72 | | 0.086 | |  |
| Proportion on stronger flow | | |  | |  | |
| Middle | -0.86 | -1.44 | | 0.15 | |  |
| Outflow | -0.36 | -0.60 | | 0.55 | |  |

*Larval feeding on leaves*

Providing an appropriate food source to *S. comalensis* in captivity is certainly very important for survival and successful reproduction. Prior to this experiment, a preliminary study was conducted using four captive-raised *S*. *comalensis* larvae to assess whether they preferred different leaf species as habitat or food sources. The limited replication in this initial study did not produce any meaningful results but informed the design of a subsequent study. The study presented below was designed to test whether there was preferential feeding on one leaf species over another in a paired system. The initial round of this study was conducted using late instar *S*. *sexlineata* larvae since they were readily obtained from the Comal Springs system. However, we were never able to obtain sufficiently more *S*. *comalensis* larvae and results from the *S*. *sexlineata* study suggest that *S*. *sexlineata* larvae have no detectible effect on leaf mass, so further study was not pursued.

In this experiment, dead leaves of three plant species commonly found around Landa Lake were collected from terrestrial habitats: *Platanus occidentalis* (American sycamore), *Ungnadia speciosa* (Mexican buckeye), and *Quercus fusiformis* (Texas live oak). Leaves were cut into 2 cm × 2 cm squares (avoiding any major veins and the midrib) and autoclaved at 121°C. After autoclaving, leaves were weighed (analytical balance with 0.0001 g accuracy) and randomly assigned to experimental chambers (10 cm × 10 cm × 15 deep). Leaves were arranged in a square, with 0.5 cm between leaves. One small (~0.5 cm diameter) limestone rock was placed on top of each leaf to hold in place so that individual leaves could be tracked across the experiment. The experimental chambers were filled with continuously flowing water from the Edwards Aquifer. Leaves were allowed to condition and develop biofilm for two weeks before larvae were added. Larvae were randomly assigned to chambers with one larva per chamber; controls had no larvae. There were six replicate experimental chambers of each species pair with larvae and two replicates of each species pairs that were controls (24 total chambers measured on two dates).

After an additional two weeks, one leaf of each species was removed from each container (two adjacent leaves were randomly selected). Leaves were air dried for at least 48 hours and then weighed. After a further two weeks, the remaining leaves were removed, dried, and weighed. All larvae were removed and returned to a separate housing chamber. We analyzed the proportional change in the mass of each leaf using a mixed effects model with the leaf species, leaf pair, larva presence, and the interaction between species and presence as fixed effects with time and experimental chamber as random effects. Exploratory analyses showed no differences across time for any species, so it was only included as a random effect.

The results showed only a significant effect of leaf species on the proportional change in mass (Table S3), with sycamore losing 56% of their mass on average, buckeye 25%, and oak 9% (Fig. S4). There were no differences in mass changes between species pairs or based on the presence/absence of larvae.

**Table S3.** Results of the mixed effect model on proportional change in leaf mass over the duration of the leaf consumption experiment. Bold indicates statistical significance (*P* < 0.05).

|  | df | *F* | *P* |
| --- | --- | --- | --- |
| Species | 2, 88 | 35.2 | **<0.0001** |
| Larva presence | 1, 88 | 0.48 | 0.49 |
| Leaf pair | 2, 88 | 1.32 | 0.27 |
| Species:Presence | 2, 88 | 1.03 | 0.36 |


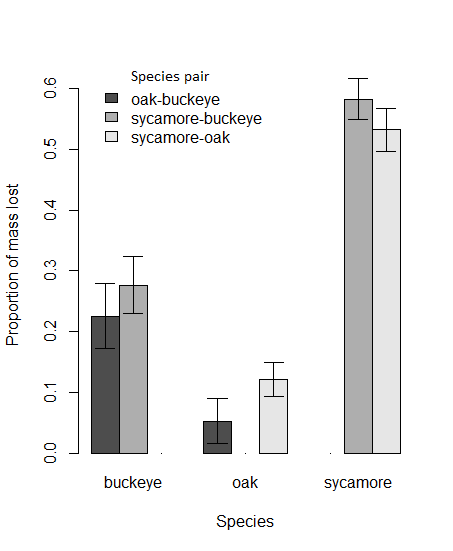


**Fig. S4.** Proportion of the leaf starting mass that was lost in the leaf consumption experiment (mean ± SE). Because neither time nor larval presence had effects in analyses, totals for each species-by-species pair are presented.
